# Genomics of Post-Prandial Lipidomic Phenotypes in the Genetics of Lipid Lowering Drugs and Diet Network (GOLDN) Study

**DOI:** 10.1371/journal.pone.0099509

**Published:** 2014-06-06

**Authors:** Marguerite R. Irvin, Degui Zhi, Stella Aslibekyan, Steven A. Claas, Devin M. Absher, Jose M. Ordovas, Hemant K. Tiwari, Steve Watkins, Donna K. Arnett

**Affiliations:** 1 Department of Epidemiology, School of Public Health, University of Alabama at Birmingham, Birmingham, Alabama, United States of America; 2 Department of Biostatistics, School of Public Health, University of Alabama at Birmingham, Birmingham, Alabama, United States of America; 3 HudsonAlpha Institute for Biotechnology, Huntsville, Alabama, United States of America; 4 Department of Epidemiology, Atherothrombosis and Imaging, Centro Nacional de Investigaciones Cardiovasculares, Madrid, Spain; 5 Instituto Madrileño de Estudios Avanzados Alimentacion, Madrid, Spain; 6 Jean Mayer USDA Human Nutrition Research Center on Aging, Tufts University, Boston, Massachusetts, United States of America; 7 Metabolon, Lipomics Division, Research Triangle Park, North Carolina, United States of America; Instituto de Investigación Sanitaria INCLIVA, Spain

## Abstract

**Background:**

Increased postprandial lipid (PPL) response to dietary fat intake is a heritable risk factor for cardiovascular disease (CVD). Variability in postprandial lipids results from the complex interplay of dietary and genetic factors. We hypothesized that detailed lipid profiles (eg, sterols and fatty acids) may help elucidate specific genetic and dietary pathways contributing to the PPL response.

**Methods and Results:**

We used gas chromatography mass spectrometry to quantify the change in plasma concentration of 35 fatty acids and 11 sterols between fasting and 3.5 hours after the consumption of a high-fat meal (PPL challenge) among 40 participants from the GOLDN study. Correlations between sterols, fatty acids and clinical measures were calculated. Mixed linear regression was used to evaluate associations between lipidomic profiles and genomic markers including single nucleotide polymorphisms (SNPs) and methylation markers derived from the Affymetrix 6.0 array and the Illumina Methyl450 array, respectively. After the PPL challenge, fatty acids increased as well as sterols associated with cholesterol absorption, while sterols associated with cholesterol synthesis decreased. PPL saturated fatty acids strongly correlated with triglycerides, very low-density lipoprotein, and chylomicrons. Two SNPs (rs12247017 and rs12240292) in the sorbin and SH3 domain containing 1 (*SORBS1*) gene were associated with b-Sitosterol after correction for multiple testing (*P*≤4.5*10^−10^). *SORBS1* has been linked to obesity and insulin signaling. No other markers reached the genome-wide significance threshold, yet several other biologically relevant loci are highlighted (eg, *PRIC285*, a co-activator of PPARa).

**Conclusions:**

Integration of lipidomic and genomic data has the potential to identify new biomarkers of CVD risk.

## Introduction

Hypertriglyceridemia and mixed dyslipidemia (i.e., high levels of low-density lipoprotein cholesterol (LDL-C) and triglycerides (TG) combined with decreased levels of high-density lipoprotein cholesterol (HDL-C)) are important in the development and progression of atherosclerosis [1]. Most clinical lipid measures continue to be assessed in the fasting state despite the fact that humans spend a considerable amount of time in a non-fasting, postprandial state and experience continuous fluctuations in the degree of lipemia throughout the day [2]. Large population-based studies have shown that delayed elimination of postprandial lipids increases cardiovascular disease (CVD) risk [3], [4], [5]. For example, the Copenhagen City Heart Study reported increasing levels of non-fasting cholesterol and non-fasting TGs were similarly associated with increasing risk of myocardial infarction over 31 years of follow-up, with non-fasting TGs being the strongest predictor in women and non-fasting cholesterol the strongest predictor in men [6].

An increased postprandial lipid (PPL) response may be inherited [7], [8]. In one report, postprandial TG levels were compared in healthy sons of men with angiographic evidence of severe coronary heart disease (CHD) versus sons of control subjects without CHD after the consumption of a high-fat liquid drink [7]. Results showed fasting lipids were comparable between groups, but the sons of men with CHD had significantly higher plasma TG levels after 8, 10, and 12 hours postprandially, suggesting delayed clearance. Additionally, our own work in GOLDN estimated the slope of TG increase 3.5 hours after a postprandial intervention had close to 80% heritability. Despite promising hypotheses (e.g., remnant triglyceride rich lipoproteins cause endothelial dysfunction), the pathogenesis of a prolonged postprandial response remains incompletely understood [2], [9]. A deeper understanding of the environmental and genetic factors influencing the PPL response will be important in developing behavioral and pharmacologic approaches to CVD prevention and treatment.

To date, studies seeking to identify genetic predictors of the PPL response in free-living populations can claim qualified success. A number of studies have documented that variants in genes such as *APOA1*, *A2*, *A4*, *A5*, *E*, *C1*, *C3*; *LPL*; *LIPC*; *SCARB1*; *MTP, GCKR*; and those encoding fatty acid binding and transport proteins, explain some of the inter-individual variability [2], [10], [11]. Despite progress, current findings do not fully explain the heritable component of the PPL response. The lipidome, as a subset of the metabolome, may offer more precise postprandial phenotypes than clinically measured lipid species. Previous studies have suggested lipidomic profiles are both heritable and under strong genetic control [12], [13]. We hypothesize that a controlled diet intervention enriched for saturated fat and cholesterol will allow more precise assessment of lipidomic postprandial changes [14] and will facilitate genetic and epigenetic discovery. The current study measured sterols and total fatty acid content across lipid classes in human plasma after a PPL intervention as part of the Genetics of Lipid-lowering Drugs and Diet Network Study (GOLDN). Genome-wide association study (GWAS) data and epigenome-wide association study (EWAS) data assayed using the Affymetrix 6.0 and the Illumina Methyl450 array, respectively, were associated with newly captured phenotypes before and after the high fat meal. Previous studies have identified correlations between genetic variation and the quantitative trait of DNA methylation enriched at nearby loci (cis-meQTLs) [15]. To interpret the potential for functional crosstalk between DNA sequence variants and methylation marks, we also examined cis-meQTLs in regions with overlapping GWAS and EWAS association.

## Methods

### Ethics Statement

The study protocol was approved by the Institutional Review Boards at the University of Minnesota, University of Utah, Tufts University/New England Medical Center, and the University of Alabama at Birmingham. Written informed consent was obtained from all participants.

### Study population

The GOLDN study was designed to identify genes that determine response of lipids to two interventions, one to raise (ingestion of high-fat meal) and one to lower lipid levels (fenofibrate treatment) [16] (registered at clinicaltrails.gov, number NCT00083369; URL, http://clinicaltrials.gov/ct2/show/NCT00083369). The GOLDN study has been previously described in Irvin *et al*. [17]. Briefly, the study ascertained and recruited families from the Family Heart Study at two centers, Minneapolis, MN and Salt Lake City, UT, who were self-reported to be white. In each case, only families with at least two siblings were recruited and only participants who did not take lipid-lowering agents (pharmaceuticals or nutraceuticals) for at least 4 weeks prior to the initial visit were included. A total of 1048 GOLDN participants were included in the diet intervention. For the current study, sterols and fatty acids were measured from stored plasma (−80 degrees Celsius) collected at fasting and 3.5 hours after the diet intervention (described below) for 40 GOLDN participants from 24 independent families, for whom EWAS data and GWAS data were available. The 3.5 hour time point was chosen for this study as it maximized the postprandial differences observed between individuals in our data facilitating genomic discovery. The phenotype and genotype data relevant to this study was deposited in The database of Genotypes and Phenotypes (dbGaP) with accession number phs000741.v1.p1.

### Postprandial intervention and clinical measurements

For the GOLDN diet intervention, participants were asked to fast for ≥12 hours and abstain from alcohol intake for ≥24 hours before visiting the clinic. The PPL intervention followed the protocol of Patsch *et al*. [18]. The whipping cream/dry milk meal had 700 calories/m^2^ body surface area (2.93 MJ/m^2^ body surface area): 3% of calories were derived from protein (instant nonfat dry milk), 14% from carbohydrate (sugar), and 83% from fat sources (heavy whipping cream). The ratio of polyunsaturated to saturated fat was 0.06 and the cholesterol content of the average meal was 240 mg. The mixture was blended with ice and 15 mL of chocolate- or strawberry-flavored syrup to increase the palatability. Blood samples were drawn immediately before (fasting) and at 3.5 and 6 hours after consuming the high-fat meal. At each of the three time points TGs were measured by glycerol-blanked enzymatic method. Cholesterol was measured using a cholesterol esterase–cholesterol oxidase reaction. The same reaction was also used to measure HDL-C after precipitation of non-HDL-C with magnesium/dextran. LDL-C was measured by a homogeneous direct method. We also used nuclear magnetic resonance (NMR) spectroscopy to measure very low-density lipoprotein cholesterol (VLDL-C) and chylomicrons. TGs, HDL-C, LDL-C, VLDL-C, chylomicrons and total cholesterol as described are referred to as clinical lipids in the context of this study. The remaining clinical measures were captured only once at fasting (or baseline) and include high-sensitivity C-reactive protein (hsCRP) measured on the Hitachi 911 using a latex particle enhanced immunoturbidimetric assay (Kamiya Biomedical Company, Seattle, WA, USA). Interleukin-6 (IL6), IL-2 soluble receptor (IL2sR)-α, tumor necrosis factor (TNF)-α, and monocyte chemoattractant protein-1 (MCP1) were measured using quantitative sandwich enzyme immunoassay techniques (ELISA kit assays, R&D Systems Inc., Minneapolis, MN, USA) as described in previous publications [19], [20]. Plasma adiponectin and insulin were measured by a commercial kit using competitive RIA (Linco Research, St Charles, MO, USA). Plasma glucose was determined by a hexokinase-mediated reaction on the Hitachi commercial kit (Roche Diagnostics). We measured weight with a beam balance, hip circumference at maximal hip girth, and waist circumference at the umbilicus. Body mass index (BMI) was calculated as weight (kg)/height (m)^2^.

### TrueMass sterol panel

Deuterium-labeled internal standards were added to 25 mL of stored plasma from blood drawn at 0 and 3.5 hours and the mixture was saponified in ethanolic KOH. Free sterols were extracted in hexane:ethanol, dried under nitrogen, and derivatized with Tri-Sil in decane. The silated free sterols were injected onto a 6890/5975 GC/MS (Agilent Technologies, CA) with a DB-5MS UI column (Agilent Technologies, CA) with helium as the carrier. Mass spectroscopic analysis was performed in the single ion monitoring (SIM) mode with electron ionization. Prior to each assay the instruments were calibrated including analysis of reference standards. Quality control (QC) samples were included with each batch to continuously monitor the accuracy of the platform with time. The absolute concentration of each sterol was determined by comparing the peak to that of the relevant internal standard. A total of 11 sterols were quantified in nmols/gram of sample including total cholesterol, 7-dehydrocholesterol, desmosterol, lanosterol, lathasterol, cholestanol, coprostanol, b-sitosterol, campesterol, stigmasterol, and 7a-hydroxycholesterol (Lipomics, West Sacramento, CA).

### TrueMass fatty acid panel

Lipids were extracted from 25 mL of stored plasma from blood drawn at 0 3.5 hours in the presence of authentic internal standards by the method of Folch *et al*. [21]. The total lipid extract was trans-esterified in sulfuric acid/methanol to create fatty acid methyl esters (FAME). The FAME were extracted into hexane and prepared for gas chromatography. Individual fatty acids were separated and quantified by capillary gas chromatography (Agilent Technologies model 6890) equipped with a 30 m HP-88 capillary column (Agilent Technologies) and a flame-ionization detector. Instruments were calibrated and QC samples were included in each batch to continuously monitor the accuracy of the platform with time. The absolute concentration of each fatty acid in the original sample was determined by comparing the peak area to that of the internal standard. A total of 35 fatty acids were quantified in nmols/gram of sample inlcuding myristic acid (14∶0); pentadecanoic acid (15∶0); palmitic (16∶0) acid; stearic acid (18∶0); arachidic acid (20∶0); behenic acid (22∶0); lignoceric acid (24∶0); myristoleic acid (14:1n5); palmitoleic acid (16:1n7); palmitelaidic acid (t16:1n7); oleic acid (18:1n9); elaidic acid (t18:1n9); vaccenic acid (18:1n7); linoleic acid (18:2n6); g-linolenic acid (18:3n6); a-linolenic acid (18:3n3); stearidonic acid (18:4n3); eicosenoic acid (20:1n9); eicosadienoic acid (20:2n6); mead acid (20:3n9); di-homo-g-linolenic acid (20:3n6); arachidonic acid (20:4n6); eicsoatetraenoic acid (20:4n3); eicosapentaenoic acid (20:5n3); erucic acid (22:1n9); docosadienoic acid (22:2n6); adrenic acid (22:4n6); docosapentaenoic acid (22:5n6); docosapentaenoic acid (22:5n3); docosahexaenoic acid (22:6n3); nervonic acid (24:1n9); and plasmalogen derivatives of 16∶0, 18∶0, 18:1n9, and 18:1n7 (Lipomics, West Sacramento, CA).

### Genotyping

DNA extraction and purification in the GOLDN study using commercial Puregene reagents (Gentra System, Inc, Minneapolis, MN), following the manufacturer's instructions has been described in Irvin *et al*. [17]. A total of 906,600 SNPs were genotyped and called using the Birdseed calling algorithm [22]. Comprehensive QC procedures excluded SNPs that were monomorphic (55,530) or had a call rate of less than 96% (82,462). In addition, SNPs were excluded from the analysis on the basis of Mendelian errors (45,778), failing the Hardy–Weinberg equilibrium test at *P*<1.0*10^−6^ (748) or minor allele frequency (MAF) <1% (63,908). Among the larger GOLDN population, SNPs passing QC were used to impute untyped SNPs using MaCH software (Version 1.0.16) with HapMap Phase II (release 22, Human Genome build 36) as the reference. Further QC excluded SNPs with discrepant alleles in comparison to mlinfo in MaCH and missing strand information. After the imputation, we created a hybrid dataset that included 2,543,887 SNPs, of which 584,029 were initially genotyped in the GOLDN population. Missing genotyped data were kept as missing in the final data set. To avoid false positive effects from associations based on very small numbers, we limited the current analysis to SNPs with MAF ≥5% for a total of 2,160,736 SNPs.

### Epigenotyping

Key genes in lipid metabolism are expressed in lymphocytes (e.g., peroxisome proliferator activated receptor a (*PPARA* [23], [24], [25])) and CD4+ T-cells were harvested from stored lymphocytes (collected at fasting) using antibody-linked Invitrogen Dynabeads [26]. We lyzed cells captured on the beads and extracted DNA using DNeasy kits (Qiagen, Venlo, Netherlands). The Illumina Infinium Human Methylation450 Beadchip was used to assess ∼470,000 autosomal CpG sites across the genome [27]. Our methods have been extensively described in Absher *et al*. [28]. For each assay, 500 ng of DNA was treated with sodium bisulfite (Zymo EZ DNA) prior to standard Illumina amplification, hybridization, and imaging steps. The resulting intensity files were analyzed with Illumina's GenomeStudio, which generated beta scores (proportion of total signal from the methylation specific probe or color channel) and “detection *P*-values” (probability that the total intensity for a given probe falls within the background signal intensity). Beta scores with an associated detection *P*-value greater than 0.01 were removed and samples with more than 1.5% missing data points were eliminated from further analysis. Furthermore, any CpG probes where more than 10% of samples failed to yield adequate intensity were removed. The filtered beta scores were then subjected to batch normalization with the ComBat package for R software in non-parametric mode (http://www.bu.edu/jlab/wp-assets/ComBat/Abstract.html). After quality control, we had data for 461,281 CpGs. Residual cell type impurities may confound epigenetic association studies.[29] Therefore, principal components based on the beta scores of all autosomal CpGs passing QC were generated and have been modeled in EWAS analyses to account for residual T-cell impurities and other technical artifacts [28]. Principal components based on the beta scores of all autosomal CpGs passing QC were generated using the *prcomp* function in R (V 2.12.1).

### Analysis

Change in each sterol and fatty acid concentration was calculated as the difference between the 3.5 hour and baseline concentrations. Statistical significance was evaluated by a test of whether the intercept associated with change was different from zero after adjustment for a random effect of family id using a linear mixed model. Pearson correlation coefficients were calculated between each sterol, fatty acid, and clinical measurements at fasting and for change with PPL. A clusterogram of fasting and postprandial correlation coefficients was constructed using the heatmap.2 function in the gplots package in R [30]. Next, we evaluated the additive effect of 2,160,736 SNPs on each fasting sterol and fatty acid concentration using mixed models adjusted for age, sex, center, and a random effect of family relationship. GWAS models implemented to evaluate postprandial sterols and fatty acids were similar but additionally adjusted for fasting concentration. We have previously reported this population to be very genetically homogeneous, and, therefore, we did not adjust for population substructure [20], [31]. For the epigenome-wide analysis, each individual sterol and fatty acid was regressed on the methylation beta score at each of the 461,281 sites adjusting for age, sex, center, and the first 4 principal components (generated to capture T-cell impurity) as fixed effects and a random effect of family. We chose 4 PCs based upon the eigenvalues and scree plot of 20 estimated PCs based on methylation data of the larger GOLDN population. Postprandial EWAS models additionally included adjustment for fasting sterol or fatty acid concentration. EWAS and GWAS models were implemented in the R *kinship* package (*lmekin* function) [32].

GWAS and EWAS analyses generated a large number of hypotheses requiring careful corrections for multiple testing. Given observed correlations among the lipidomic phenotypes we used matrix spectral decomposition [33] as a data reduction technique to estimate the number of independent sterol phenotypes (9 of 11 were independent) and the number of independent fatty acid phenotypes (17 of 35 were independent). Using the most conservative approach (the Bonferroni correction) for GWAS, alpha (a) was set to 0.05/(2,160,736*9) = 2.6*10^-9^ for sterols, and, likewise, a = 0.05/(2,160,736*17) = 1.4*10^−9^ for fatty acids. Correction for multiple testing during EWAS followed similar logic (a = 1.2*10^−8^ and a = 6.4*10^−9^ for sterols and fatty acids, respectively). SNPs and CpGs with association *P*-value <1.0*10^−4^ were annotated using the UCSC genome browser and ANNOVAR [34], [35].

The UCSC Batch Coordinate Conversion (liftOver) module was used to convert genome coordinates of SNPs from hg18 to hg19 similarly as CpG sites. Any regional overlap (within 20 kb) of EWAS and GWAS signals of at least marginal significance (defined as *P*<1.0*10^−4^) was identified. SNP association with methylation beta score was examined (cis-meQTL association) in regions of overlap adjusting for covariates (age, sex, center, methylation data PCs) as fixed effects and family structure as a random effect.

## Results

**Figure 1:**
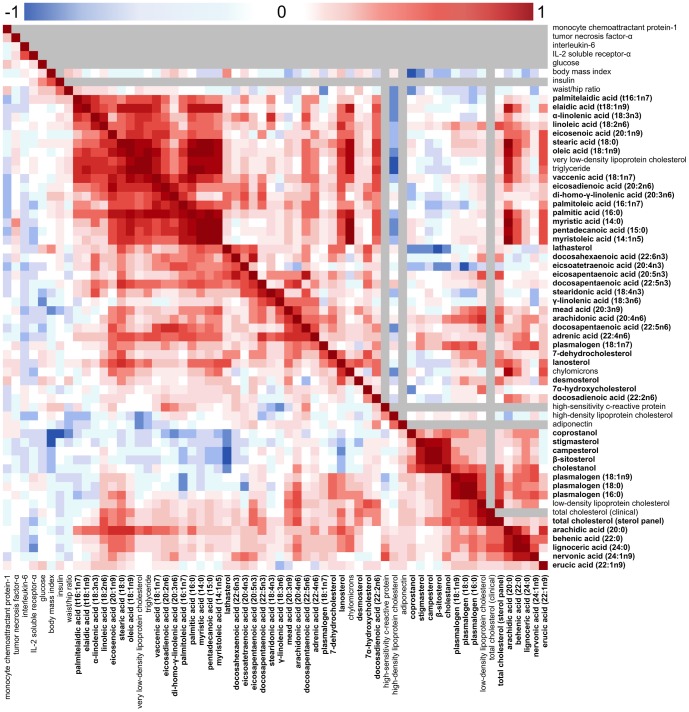
Data from 40 Genetics of Lipid Lowering Drugs and Diet Network Study participants. 1a (below the diagonal)-Pairwise correlation of fasting sterols (bold), fatty acids (bold), clinical lipids, inflammatory markers, and other clinical measures. 1b (above the diagonal)- Pairwise correlation of change in postprandial sterols (bold), fatty acids (bold), clinical lipids, and other clinical measures. Grey lines indicate clinical parameters not captured postprandially.

Participants were on average 51.2±13 years old, 55% were male, and 60% were recruited from the GOLDN Minneapolis field center. For a comparison of clinically measured lipids at fasting and 3.5 hours after the intervention and other relevant variables measured in GOLDN among the 40 participants see Table S1. Mean concentration for each of the 11 sterols and 35 fatty acids are presented in Table 1. The average fasting values of each metabolite were within the normal range set by TrueMass internal standards and representative of the assay's typical performance. On average campesterol, b-sitosterol, desmosterol, coprostanol, and cholestanol increased 3.5 hours after the high-fat diet intervention and lathosterol and lanosterol decreased. Stigmasterol, 7-dehydrocholesterol, and 7a-hydroxycholesterol did not significantly change. Overall, total plasma fatty acids increased postprandially (Table 1). Pairwise correlations between fasting clinical measures, inflammatory markers, and newly captured fatty acids and sterols are presented in Figure 1a. In the fasting state, inflammatory markers, BMI, insulin and glucose did not strongly correlate with clinical lipids, fatty acids, or sterols. Fatty acids were strongly and positively correlated with each other in addition to TG and VLDL-C. The strongest correlations observed were among TG, VLDL-C, oleic acid and vaccenic acid. LDL-C and total cholesterol moderately correlated with plasmalogens and longer chain saturated fatty acids. Sterols, including stigmasterol, campesterol, b-sitosterol, and cholestanol, were strongly and positively correlated in the fasting state while lanosterol and lathasterol were negatively correlated with those sterols. Figure 1b shows pairwise correlations among the changes in PPL clinical lipids, fatty acids, and sterols. Many of the observed trends were consistent with that observed at fasting or were stronger, particularly saturated fatty acids (palmitic acid, pentadecanoic acid, myristic acid, and stearic acid) and their biosynthetic products (myristoleic acid, vaccenic acid, oleic acid, and elaidic acid) were strongly correlated with VLDL-C and TG. In the postprandial state, change in chylomicrons positively correlated with that group while change HDL-C negatively correlated with that group.

**Table 1:** Average concentration (nmol/gram of sample) of TrueMass panel metabolites measured at fasting and 3.5 hours after a postprandial lipemia (PPL) challenge for 40 Genetics of Lipid Lowering Drugs and Diet Network Study participants.

TrueMass Panel Metabolites	Fasting	3.5 hrs	Direction*	P-value
*Sterols*				
total cholesterol	4388.86±617.2	4561.12±599.6	Up	0.0006
7-dehydrocholesterol	6.32±2.3	6.56±2.5	Up	0.2038
desmosterol	3.45±1.1	3.62±1.2	Up	0.0001
lanosterol	0.42±0.2	0.38±0.1	Down	0. 0012
lathasterol	13.99±4.7	13.47±5.4	Down	0.0484
cholestanol	8.91±2.3	9.21±2.1	Up	0.0101
coprostanol	0.08±0.05	0.09±0.05	Up	0.0017
b-sitosterol	11.20±5.2	11.86±4.7	Up	<0.0001
campesterol	6.38±3.1	6.83±3.0	Up	<0.0001
stigmasterol	0.48±0.2	0.51±0.2	Up	0.2049
7a-hydroxycholesterol	0.94±0.4	0.90±0.4	Down	0.2443
*Fatty acid*				
myristic acid (14∶0)	206.86±88.8	934.74±371.0	Up	0.0003
pentadecanoic acid (15∶0)	40.97±11.1	111.60±35.1	Up	<0.0001
palmitic acid (16∶0)	3367.23±769.7	5715.29±1369.6	Up	<0.0001
stearic acid (18∶0)	1062.27±198.1	1942.90±502.7	UP	<0.0001
arachidic acid (20∶0)	11.62±3.21	20.73±4.76	Up	<0.0001
behenic acid (22∶0)	20.03±7.83	21.68±5.94	Up	0.0411
lignoceric acid (24∶0)	17.80±7.41	19.29±5.68	Up	0.1372
myristoleic acid (14:1n5)	17.33±9.68	101.82±28.58	Up	<0.0001
palmitoleic acid (16:1n7)	318.60±127.4	462.49±155.0	Up	<0.0001
vaccenic acid (18:1n7)	235.01±59.53	326.19±68.81	Up	<0.0001
oleic acid (18:1n9)	3181.79±830.1	4942.18±1322.7	Up	<0.0001
eicosenoic acid (20:1n9)	22.06±6.32	27.94±6.91	Up	<0.0001
mead acid (20:3n9)	17.53±5.7	20.20±7.0	Up	<0.0001
erucic acid (22:1n9)	5.18±1.13	7.15±6.37	Up	0.0645
nervonic acid (24:1n9)	13.50±3.49	13.63±2.24	Up	0.7983
linoleic acid (18:2n6)	4574.14±725.4	5254.04±789.1	Up	<0.0001
γ-linolenic Acid (18:3n6)	80.212±24.5	91.29±28.6	Up	<0.0001
eicosadienoic acid (20:2n6)	38.87±9.59	49.15±10.09	Up	<0.0001
di-homo-γ-linolenic Acid (20:3n6)	238.18±56.3	268.53±58.2	Up	<0.0001
arachidonic acid (20:4n6)	1081.37±216.2	1207.81±236.2	Up	<0.0001
docosadienoic acid (22:2n6)	1.41±0.60	1.60±0.48	Up	0.2269
adrenic acid (22:4n6)	37.53±8.24	45.22±7.70	Up	<0.0001
docosapentaenoic acid (22:5n6)	28.78±9.31	32.36±10.04	Up	<0.0001
a-linolenic acid (18:3n3)	95.03±32.38	136.67±34.94	Up	<0.0001
stearidonic acid (18:4n3)	4.82±2.18	6.17±2.26	Up	<0.0001
eicsoatetraenoic acid (20:4n3)	12.09±5.38	14.98±4.36	Up	0.001
eicosapentaenoic acid (EPA) (20:5n3)	69.79±24.61	80.86±27.91	Up	<0.0001
docosapentaenoic acid (DPA) (22:5n3)	77.76±19.18	94.32±21.01	Up	<0.0001
docosahexaenoic acid (DHA) (22:6n3)	202.66±61.02	228.33±67.78	Up	<0.0001
plasmalogen (16∶0)	41.60±8.01	44.47±9.28	Up	0.0003
plasmalogen (18∶0)	33.18±8.37	35.97±9.41	Up	0.002
plasmalogen (18:1n7)	2.04±0.97	2.14±1.02	Up	0.5892
plasmalogen (18:1n9)	9.25±2.51	10.32±2.19	Up	0.001
palmitelaidic acid (t16:1n7)	53.03±16.37	73.26±23.01	Up	<0.0001
elaidic acid (t18:1n9)	216.25±89.87	399.28±125.89	Up	<0.0001

*direction of change in concentration of the metabolite after the PPL intervention.

**Figure 2:**
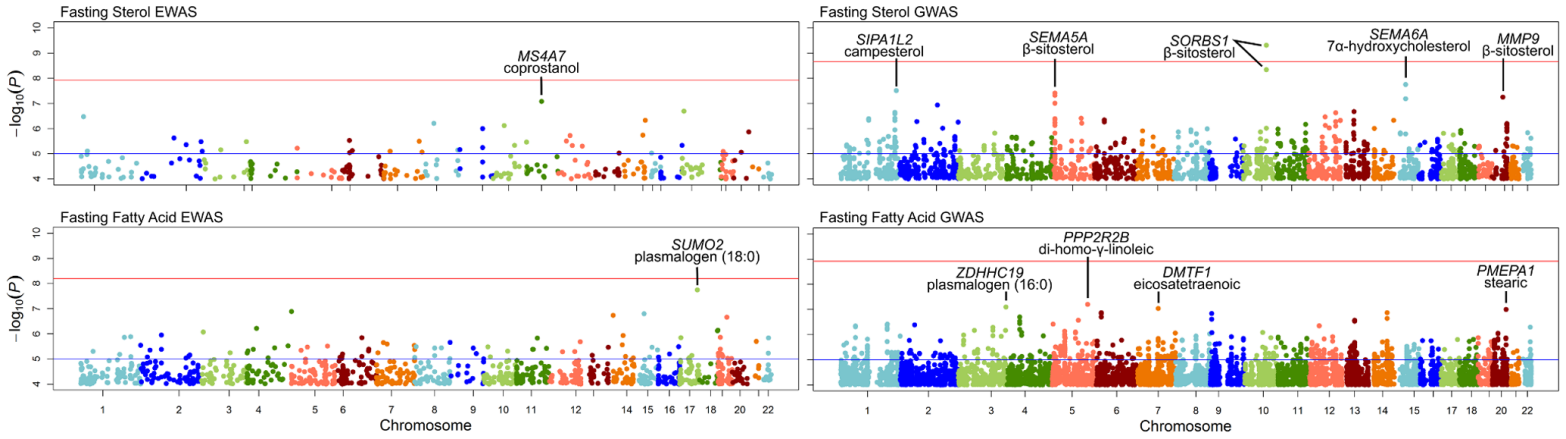
Manhattan plots for markers with *P*<0.0001 from epigenome-wide association study and genome-wide association study. Phenotypes include 11 sterols and 35 fatty acids measured at fasting.

Manhattan plots of association signals with *P*<1.0*10^−4^ from EWAS and GWAS of fasting sterols and fatty acids are presented in Figure 2. GWAS results from each of 11 sterols are combined in the upper right hand quadrant of Figure 2 and, likewise, GWAS results for each of the 35 fatty acids are combined in the bottom right hand quadrant. For a complete list of all CpGs and SNPs (including annotations) shown in Figure 2, see Spreadsheet S1. Table 2 highlights GWAS and EWAS results from analysis of fasting sterol and fatty acids with *P*<1.0*10^−7^. For sterols the strongest genetic signal (*P*<4.5*10^−9^ for each of 5 SNPs) came from a region on chromosome 10 within the sorbin and SH3 domain containing 1 gene (*SORBS1*). The top two SNPs (rs12247017 and rs12240292) met significance criteria (*P*<2.6*10^−9^) after correction for multiple testing. The second strongest signal (*P*<1.8*10^−8^ for each of 3 SNPs) was for 7a-hydroxycholesterol in the semaphorin 6D (*SEMA6D*) gene on chromosome 15. Each of the 3 SNPs lies in the first intron of an alternate transcript of *SEMA6D*. Fourteen intronic SNPs in *SEMA5D* on chromosome 5 were strongly associated with fasting b-sitosterol (*P*≤9.8*10^−8^). A SNP (rs3918278) on chromosome 20 upstream of the matrix metallopeptidase 9 (*MMP9*) gene was also associated with b-sitosterol (*P* = 5.6*10^−8^). In EWAS, no CpG was statistically significantly associated with any sterol after correction for multiple testing. Table 2 highlights a CpG (cg02621636) on chromosome 11 associated with coprostanol in the 3′ UTR of the membrane-spanning 4-domains, subfamily A, member 7 gene (*MS4A7*). Finally, no SNP or CpG was statistically significantly associated with any of the 35 fasting fatty acid concentrations considered (Figure 2). Marginally significant markers are highlighted in Table 2 and include 2 SNPs on chromosome 5 upstream of the protein phosphatase 2, regulatory subunit B, beta gene (*PPP2R2B*) associated with di-homo-γ-linoleic acid (DGLA).

**Table 2:** Top genome-wide association study (GWAS) and epigenome-wide association study (EWAS) results for fasting sterols and fatty acids.

Marker	Chr	Location	Lipid	Gene	Gene Proximity	P-value
*Sterol GWAS*						
rs12247017	10	97270729	b-sitosterol	*SORBS1*	intronic	4.9*10^−10^
rs12240292	10	97273066	b-sitosterol	*SORBS1*	intronic	4.5*10^−10^
rs12772243	10	97295589	b-sitosterol	*SORBS1*	intronic	4.5*10^−9^
rs12776555	10	97284362	b-sitosterol	*SORBS1*	intronic	4.5*10^−9^
rs4918944	10	97283881	b-sitosterol	*SORBS1*	intronic	4.5*10^−9^
rs281282	15	47679257	7a-hydroxycholesterol	*SEMA6D*	intronic	1.8*10^−8^
rs281284	15	47680407	7a-hydroxycholesterol	*SEMA6D*	intronic	1.8*10^−8^
rs281294	15	47683646	7a-hydroxycholesterol	*SEMA6D*	intronic	1.8*10^−8^
rs1631842	1	232447391	campesterol	*SIPA1L2*	∼100kb upstream	3.1*10^−8^
rs1766581	1	232447293	campesterol	*SIPA1L2*	∼100kb upstream	3.1*10^−8^
rs10059341	5	9325205	b-sitosterol	*SEMA5A*	intronic	3.9*10^−8^
rs10065505	5	9301719	b-sitosterol	*SEMA5A*	intronic	3.9*10^−8^
rs13360783	5	9299238	b-sitosterol	*SEMA5A*	intronic	3.9*10^−8^
rs1557879	5	9308539	b-sitosterol	*SEMA5A*	intronic	3.9*10^−8^
rs3777306	5	9295681	b-sitosterol	*SEMA5A*	intronic	3.9*10^−8^
rs3777311	5	9301324	b-sitosterol	*SEMA5A*	intronic	3.9*10^−8^
rs3777312	5	9301496	b-sitosterol	*SEMA5A*	intronic	3.9*10^−8^
rs3777316	5	9302667	b-sitosterol	*SEMA5A*	intronic	3.9*10^−8^
rs3777320	5	9307427	b-sitosterol	*SEMA5A*	intronic	3.9*10^−8^
rs3777325	5	9319778	b-sitosterol	*SEMA5A*	intronic	3.9*10^−8^
rs3777327	5	9323467	b-sitosterol	*SEMA5A*	intronic	3.9*10^−8^
rs3797980	5	9328316	b-sitosterol	*SEMA5A*	intronic	3.9*10^−8^
rs17196572	5	9298117	b-sitosterol	*SEMA5A*	intronic	4.7*10^−8^
rs3918278	20	44635654	b-sitosterol	*MMP9*	∼2k bupstream	5.6*10^−8^
rs11070582	15	47686590	7a-hydroxycholesterol	*SEMA6D*	intronic	6.5*10^−8^
rs3822789	5	9322477	b-sitosterol	*SEMA5A*	intronic	9.8*10^−8^
rs697651	2	155323278	7-dehydrocholesterol	*GALNT13*	downstream	1.2*10^−7^
*Sterol EWAS*						
cg02621636	11	60161999	coprostanol	*MS4A7*	in 3'utr	8.3*10^−8^
*FA GWAS*						
rs6895471	5	146472853	di-homo-γ-linoleic	*PPP2R2B*	∼10kb upstream	6.3*10^−8^
rs17105882	5	146465362	di-homo-γ-linoleic	*PPP2R2B*	∼10kb upstream	6.3*10^−8^
rs6795707	3	195937410	plasmalogen (16∶0)	*ZDHHC19*	intronic	8.0*10^−8^
rs1859124	7	86747876	eicsoatetraenoic	*DMTF1*	∼50 kb upstream	9.2*10^−8^
rs8118851	20	56292879	stearic	*PMEPA1*	∼10 kb upstream	1.0*10^−7^
*FA EWAS*						
cg23221506	17	73175571	plasmalogen (18∶0)	SUMO2	intron	1.8*10^−8^

Chr, chromosome; FA, fatty acid.

**Figure 3:**
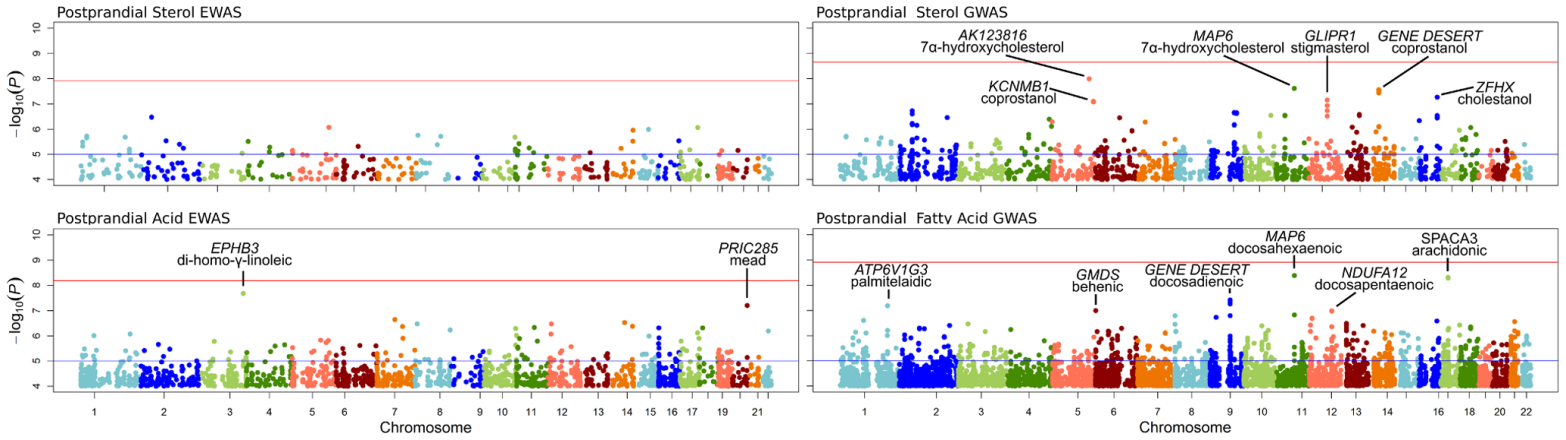
Manhattan plots for markers with *P*< 0.0001 from epigenome-wide association study and genome-wide association study. Phenotypes include 11 postprandial sterols and 35 postprandial fatty acids after adjustment for fasting concentration.

Manhattan plots of association signals with *P*<1.0*10^−4^ from EWAS and GWAS of postprandial sterols and fatty acids (adjusted for fasting concentrations) are presented in Figure 3. For a complete list of all CpGs and SNPs (including annotations) shown in Figure 3, see Spreadsheet S2. Table 3 highlights GWAS and EWAS results from the postprandial analysis of sterols and fatty acids with *P*<1.0*10^−7^. No marker in EWAS or GWAS was statistically significantly associated with any PPL sterol or fatty acid after correction for multiple testing. Marginally significant sterol GWAS signals included 5 markers on chromosome 14 associated with coprostanol that were not near (within 200kb up or downstream) any characterized gene. Four SNPs on chromosome 5 were also associated with postprandial coprostanol concentration after adjustment for fasting concentration. The region is gene rich and the 4 SNPs lie in intron 1 of an alternate transcript of the Kv channel interacting protein 1 (*KCNIP1*) gene in addition to intron 1 of a smaller overlapping gene known as potassium large conductance calcium-activated channel, subfamily M, beta member 1 (*KCNMB1*). No CpGs were associated with any postprandial sterol with *P*<1.0*10^−7^ and, thus, are not represented in Table 3. The SNP rs666566 in the microtubule-associated protein 6 (*MAP6*) gene associated with docosahexaenoic acid (DHA) was the top hit for the fatty acid GWAS. The same SNP was also associated with 7a-hydroxycholesterol. Another SNP (rs685448) in *MAP6* was associated with DHA with *P* = 1.5*10^−7^ and with 7a-hydroxycholesterol with *P* = 9.5*10^−5^. A group of SNPs (rs16843235, rs2759275, rs16843150) upstream of ATPase, H+ transporting, lysosomal 13kDa, V1 subunit *G3 (ATP6V1G3*) were associated with palmitelaidic acid. Finally, two CpGs are highlighted in Table 3 for fatty acids, the first (cg15718583) with DGLA in the EPH receptor B3 (*EPHB3*) gene and another (cg03758021) with mead acid in the PPARA interacting complex 285 (*PRIC285*) gene.

**Table 3:** Top genome-wide association study (GWAS) and epigenome-wide association study (EWAS) results for postprandial sterols and fatty acids.

Marker	Chr	Location	Lipid	Gene	Gene Proximity	P-value
*Sterol GWAS*						
rs17490390	5	152362706	7a-hydroxycholesterol	*AK123816*	∼10 kb downstream	1.0*10^−8^
rs666566	11	75302420	7a-hydroxycholesterol	*MAP6*	intronic	2.4*10^−8^
rs1627411	14	43171328	coprostanol	*-*	-	2.7*10^−8^
rs1698533	14	43172278	coprostanol	*-*	-	2.8*10^−8^
rs1698534	14	43172343	coprostanol	*-*	-	2.8*10^−8^
rs1712738	14	43163097	coprostanol	*-*	-	3.0*10^−8^
rs1627270	14	43168725	coprostanol	*-*	-	3.6*10^−8^
rs7203768	16	73301302	cholestanol	*ZFHX3*	∼200 kb downstream	5.4*10^−8^
rs12826237	12	76073557	stigmasterol	*GLIPR1*	∼100 kb downstream	7.0*10^−8^
rs314112	5	169813739	coprostanol	*KCNMB1*	intronic	7.8*10^−8^
rs314111	5	169813844	coprostanol	*KCNMB1*	intronic	8.1*10^−8^
rs314113	5	169813296	coprostanol	*KCNMB1*	intronic	8.1*10^−8^
rs314109	5	169814432	coprostanol	*KCNMB1*	intronic	8.2*10^−8^
rs17223072	12	76080856	stigmasterol	*GLIPR1*	∼100kb downstream	1.1*10^−7^
*FA GWAS*						
rs666566	11	75302420	docosahexaenoic	*MAP6*	intronic	4.1*10^−9^
rs757079	17	31325103	arachidonic	*SPACA3*	∼0.2kb downstream	4.9*10^−9^
rs28958	17	31324657	arachidonic	*SPACA3*	intronic	5.3*10^−9^
rs13285452	9	83595061	docosadienoic	*-*	-	3.9*10^−8^
rs7025679	9	83513137	docosadienoic	*-*	-	4.1*10^−8^
rs7350230	9	83572513	docosadienoic	*-*	-	4.5*10^−8^
rs7024926	9	83576272	docosadienoic	*-*	-	5.5*10^−8^
rs16843235	1	198485694	palmitelaidic	*ATP6V1G3*	∼10 kb upstream	6.5*10^−8^
rs9503012	6	1707004	behenic	*GMDS*	intronic	1.0 *10^−7^
rs1628439	12	95400638	docosapentaenoic	*NDUFA12*	∼5 kb downstream	1.0 *10^−7^
*FA EWAS*						
cg15718583	3	184231385	di-homo-γ-linoleic	*EPHB3*	∼50 kb upstream	2.1 *10^−8^
cg03758021	20	62208140	mead	*PRIC285*	∼5 kb downstream	6.3 *10^−8^

Chr, chromosome; FA, fatty acid.

We explored overlap of EWAS and GWAS results within 20 kb with *P*<1.0*10^−4^ across the genome for each sterol and fatty acid at each time point and evaluated cis-meQTL signals for highlighted regions. Results are presented in Spreadsheet S3. Five regions of EWAS/GWAS overlap were identified for the fasting analysis of sterols and fatty acids including regions on chromosomes 1, 11, 16, 19 and 21 for fasting total cholesterol, coprostanol, palmitoleic acid, docosapentaenoic acid, and adrenic acid, respectively, with evidence of moderate to strong cis-meQTL signal (5.0*10^−3^≤*P*≤5.1*10^−133^). Consideration of EWAS/GWAS overlap for postprandial models highlighted 10 regions of interest that also consist of many strong cis-meQTL signals. For instance, cg22761176 and cg26817877, located less than 100 bp apart on chromosome 2, were associated with a group of nearby SNPs with cis-meQTL association significance level ranging from *P* = 0.06 to *P* = 2.7*10^−21^.

## Discussion

In the current study we measured the concentration of 11 sterols and 35 fatty acids before and after a PPL challenge in 40 participants from the Genetics of Lipid Lowering Drugs and Diet Network study. We evaluated change in plasma sterols and fatty acids after the challenge, their correlation with other clinical measures in the fasting and postprandial state, and integrated the new lipid phenotypes with existing genomic data. Our findings demonstrate marked response of sterols and fatty acids to the PPL challenge, correlations between clinical lipids and newly measured lipids, and provide proof-of-concept that genomic studies of sterols and fatty acids may reveal new information about pathways important to lipid metabolism and ultimately help identify new biomarkers of CVD risk.

Cholesterol homeostasis, involving the balance between absorption and synthesis, influences circulating plasma lipoprotein concentrations [36], [37], [38], [39], [40]. Therefore, several studies have assessed whether cholesterol absorption and synthesis are also associated with prevalent CVD [40]. Results have varied with some studies reporting that higher cholesterol absorption and/or lower cholesterol synthesis is associated with increased [41], [42], [43], [44], [45], [46], [47], decreased [48], [49], or no difference in CVD risk [50], [51], [52]. Because cholesterol absorption and biosynthesis are difficult to measure directly, we measured circulating concentrations of several cholesterol-related sterols as biomarkers of these processes. b-Sitosterol, campesterol, and stigmasterol are plant sterols (phytosterols) similar in structure to cholesterol. Although the absolute amount of phytosterols absorbed in the gut is very low, their plasma concentrations are correlated with cholesterol absorption [53]. Cholestanol, an endogenous 5-α–reduced metabolite of cholesterol, is also correlated with cholesterol absorption [53], [54]. Lanosterol, lathosterol, 7-dehydrocholesterol, and desmosterol, are endogenous precursors of cholesterol correlated with cholesterol synthesis [54], [55]. Other metabolic products measured in this study include an oxidized derivative of cholesterol, 7a-hydroxylcholesterol, and coprostanol, a dietary cholesterol derivative produced by the intestinal microbiota [56], [57], [58], [59]. Overall, markers of cholesterol absorption significantly increased and markers of cholesterol synthesis decreased 3.5 hours after the high fat meal. Ultimately, a better understanding of the genetic background of these processes can help explain mechanisms related to postprandial lipemia and even inform future studies relating sterols to CVD risk.

Fatty acid metabolism is also intricately tied to CVD health. Increasing dietary saturated fatty acid intake increases total cholesterol, LDL-C and TG [60], [61], [62], [63]. Subsequently, reduced saturated fatty acid intake has been an important dietary recommendation for the reduction of CVD risk [64]. However, individual saturated fatty acids have unique properties, form a variety of metabolites and, thus, have diverse biological functions [65]. Importantly, not all saturated fatty acids have the same cholesterol-raising effects. For instance, stearic acid has a neutral effect on total cholesterol, LDL-C, and HDL-C, whereas lauric, myristic, and palmitic acids increase total cholesterol, LDL-C, and HDL-C, with myristic acid having the most potent hypercholesterolemic effect [60], [66], [67]. Due to the complexity of saturated fatty acids in foods, their consumption in the context of other nutrients and sources of error in dietary exposure information, the effect of individual saturated fatty acids on CVD endpoints has varied in the literature [65], [68], [69], [70], [71]. An advantage of the study set within GOLDN is that the meal has been standardized and each participant acted as his or her own control. We evaluated saturated fatty acids (myristic acid, pentadecanoic acid, palmitic acid, stearic acid, arachidic acid, behenic acid, and lignoceric acid) and their direct biosynthetic products (myristoleic acid, palmitoleic acid, vaccenic acid, oleic acid, eicosenoic acid, mead acid, erucic acid, and nervonic acid), but also essential fatty acids (a-linolenic acid, linoleic acid) and their longer, more desaturated derivatives (g-linolenic acid, eicosadienoic acid, di-homo-g-linolenic acid, arachidonic acid, docosadienoic acid, adrenic acid, docosapentaenoic acid, stearidonic acid, eicosoatetraenoic acid, eicosapentaenoic acid, docosapentaenoic acid, and docosahexaenoic acid), 4 plasmalogens and the trans fatty acids elaidic acid and palmitelaidic acids. In our small study, fatty acids present in bovine milk fat [72] (saturated fatty acids- palmitic acid, myristic acid, and stearic acid; mono-unsaturated fatty acid- oleic acid; and trans fatty acids vaccenic acid and elaidic acid) correlated most closely with TG and lipid species that carry TG in response to the PPL challenge. Yet several other fatty acid species (many derivatives of these saturated fatty acids) positively correlated with this group potentially reflecting intrinsic metabolic response to the fat load (e.g. palmitoleic acid, plamitelaidic acid, eicosenoic acid, erucic acid). Importantly, our findings support prior research correlating fatty acids with clinical lipids and provide a unique setting to evaluate the genomic determinants of inter-individual differences in specific fatty acids after a standardized PPL challenge.

GWAS and EWAS of fasting sterol and fatty acid concentrations highlighted several regions of interest with biologically plausible association. A cluster of SNPs on chromosome 10 associated with b-sitosterol is located in *SORBS1*, which encodes a protein in the insulin signaling pathway [73]. Insulin resistance has been linked to high cholesterol synthesis and decreased cholesterol absorption in prior reports [54], [74], [75]. Further studies are needed to determine if variation in *SORBS1* may help explain such observations. SNPs in *SEMA6D* and *SEMA5A* were highlighted as being associated with fasting 7a-hydroxycholesterol and b-sitosterol, respectively. Semaphorins have important regulatory functions in the cardiac, circulatory and immune systems [76], [77], [78], [79], [80]. For instance, *SEMA6D* has been linked to T-cell activation [81] and *SEMA5A* promotes angiogenesis through increased endothelial cell proliferation, migration, and downregulated apoptosis [82]. *SEMA5A* action may be facilitated by *MMP9*, another gene reported as being associated with b-sitosterol in Table 2 [82]. *MMP9* has been indicated in vascular injury, inflammation, and tissue remodeling associated with CVD [83], [84], [85], [86]. A CpG in the 3′UTR of *MS4A7* was associated with coprostanol. *MS4A7* encodes a member of the membrane-spanning 4A gene family, localized to chromosome 11q12, in a cluster of other family members. *MS4A7* is expressed in lymphocytes and is likely involved in signal transduction [87] SNPs upstream of *PPP2R2B* were associated with DGLA, an n-6 polyunsaturated fatty acid (PUFA). *PPP2R2B* encodes a brain specific regulatory subunit of a protein phosphatase that is not easily relatable to CVD and lipid metabolism. However, a recent report that fine-mapped a large region on chromosome 5q31-33 for early onset coronary artery disease (CAD) across two large family studies identified *PPP2R2B* as one of four genes that showed consistent and strong association with both LDL-C and CAD [88].

After adjusting for fasting concentration, GWAS and EWAS of individual PPL sterol and fatty acid concentrations also yielded unique results in or near genes with compelling biological significance to lipid metabolism and CVD. *ZFHX3* is a transcription factor with cardiac and immune cell expression. Though our signal for cholestanol is much farther downstream, SNPs in *ZFHX3* have been linked to stroke and atrial fibrillation in prior GWAS [89], [90], [91]. GLI pathogenesis-related 1 (*GLIPR1*) may be linked immune cell function as the gene is highly expressed in multiple immune cell types [92]. Calcium activated K+ channels (KCa) have an essential role in arterial function regulating vascular tone [93]. Our study highlights SNPs in *KCNMB1* (encoding a KCa subunit) which were associated with postprandial coprostanol. Previous research has linked mutations in *KCNMB1* to hypertension, myocardial infarction and stroke [94]. Additionally, increased expression of vascular KCa channels have been demonstrated in coronary vessels from patients with CAD [95]. GDP-mannose-4,6-dehydratase (GMDS), highlighted for association with behenic acid, is regulator of post-translational modification processes important for cell surface lipids and proteins [96]. Other genes annotated in GWAS are more difficult to link to postprandial lipid metabolism. For instance, MAP6 is involved in microtubule stabilization in many cell types, and sperm acrosome associated 3 (SPACA3) is a sperm surface membrane protein [92], [97], [98]. Finally, fatty acid EWAS results highlight a marker (cg03758021) closely downstream of *PRIC285*, a nuclear co-activator of the transcription factor PPARA which is intricately involved inflammation and lipid metabolism in the liver [99], [100].

In addition to evaluating the most significant EWAS and GWAS results in this study we mined all results presented in Spreadsheets S1 and S2 for postprandial lipemia and cardiovascular risk loci published in the literature [2], [10], [11], [101]. A SNP (rs219562) near *APOB* was associated with postprandial stigmasterol (*P* = 6.0*10^−5^) and a CpG (cg20691580) in *APOC3* was associated with postprandial palmitelaidic acid (*P* = 3.0*10^−5^). *APOB* and *APOC3* have been associated with postprandial clinical lipids [2]. Additionally, CpGs in *WDR12* and *SH2B3*, respectively, were found in Spreadsheet S1 among the fasting fatty acid EWAS results. Finally, a SNP (rs11239204) near *CXCL12* was associated (*P* = 1.8*10^−5^) with postprandial plasmalogen 18:1n9 concentration. *WDR12*, *SH2B3* and *CXCL12* were among 13 loci highlighted in a large GWAS of CAD [101]. Though none of these findings meet criteria for statistical significance after correction for multiple testing, they support the potential usefulness of lipidomic studies in unraveling biological mechanisms by which genes relate to more complex CVD phenotypes.

We also explored the potential to integrate EWAS and GWAS results from these analyses. Taking a regionalized approach our results demonstrate several examples of within phenotype EWAS/GWAS overlap in both fasting and postprandial models. In the future, we hope to have data from a larger sample in GOLDN that will enable us to model SNPs and CpG sites (fitting criteria as cis-meQTLs) jointly on a phenotype to better deduce interplay among markers. While our small sample restricted the pursuit of these additional analyses, our data demonstrate the potential for functional crosstalk between genomic layers supporting further expansion of this research set within GOLDN.

We note several limitations to our study. Most importantly, our sample size was small for the high dimensional data analyses pursued. Therefore, it was difficult to identify markers meeting the strict criteria necessary to achieve statistical significance in the context of this study. Consequently, some false positive and alternatively, false negative findings may have occurred. Replication in a larger group from GOLDN and external cohorts is needed. Still, we present novel research that highlights the potential of integrating ‘omic’ data to make progress in the field of translational research and personalized medicine in the context of an altered postprandial lipid response.

To our knowledge, this is the first study evaluating the lipid response to a PPL challenge with fine resolution of phenotypes via lipidomic assays. Additionally, a new dimension to the study of postprandial lipemia was explored by integrating GWAS and EWAS data with the lipidomic measurements. Our study highlighted several novel genes involved in lipid metabolism, endothelial function, immune function and cell signaling. Overall, the results demonstrate that small molecule lipids correlate with clinical lipids, respond to the PPL intervention, and that genomic markers might help unravel mechanisms related to lipid metabolism in the fasting and non-fasting state. This report sets the groundwork to expand this research in GOLDN and other cohorts to help translate novel biomarkers of the postprandial lipid response to use in the diagnosis, prevention and treatment of dyslipidemias and CVD.

## Supporting Information

Table S1 **Clinical variables measured at fasting and 3.5 hours after the postprandial lipemia challenge in 40 Genetics of Lipid Lowering Drugs and Diet Network (GOLDN) study participants.**
(PDF)

Spreadsheet S1 **Complete list of all CpGs and SNPs (including annotations) shown in** **Figure 2** **.**
(XLS)

Spreadsheet S2 **Complete list of all CpGs and SNPs (including annotations) shown in** **Figure 3** **.**
(XLS)

Spreadsheet S3 **cis-MeQTL association in regions of EWAS/GWAS overlap.**
(XLSX)
