# Supplementary material for: Genomics of Post-Prandial Lipidomic Phenotypes in the Genetics of Lipid Lowering Drugs and Diet Network (GOLDN) Study
Source: PLoS One. 2014 Jun 6;9(6):e99509. doi: 10.1371/journal.pone.0099509 (PMC4048279; doi:10.1371/journal.pone.0099509)
Supplement: Table S1 — Clinical variables measured at fasting and 3.5 hours after the postprandial lipemia challenge in 40 Genetics of Lipid Lowering Drugs and Diet Network (GOLDN) study participants. (PDF) [file pone.0099509.s001.pdf]

Supplemental Table 1: Clinical variables measured at fasting and 3.5 hours after the postprandial lipemia challenge in 40 Genetics of Lipid Lowering Drugs and Diet Network (GOLDN) study participants

**Clinical Measurement**	**Fasting**	**3.5 hours***
VLDL-C (total) (mg/dL)	156.32 ± 73.6	306.25 ± 113.4
Chylomicrons (mg/dL)	5.70 ± 5.1	123.93 ± 76.1
LDL-C (mg/dL)	132.72 ± 26.3	137.68 ± 29.1
HDL-C (mg/dL)	38.69 ± 10.3	37.35 ± 10.8
Triglycerides (mg/dL)	185.35 ± 80.7	412.75 ± 145.8
Total Cholesterol(mg/dL)	209.75 ± 29.0	-
Glucose (mg/dL)	100.75 ± 14.9	-
Insulin (mU/L)	13.70 ± 7.6	-
BMI (kg/m2)	29.02 ± 3.7	-
Waist/Hip Ratio	0.93 ± 0.06	-
IL-6 (pg/mL)	2.09 ± 2.9	-
hsCRP (mg/dl)	0.18 ± 0.2	-
MCP-1 (pg/mL)	216.38 ± 63.3	-
TNF-a (pg/mL)	4.21 ± 4.2	-
IL-2 sRa (pg/mL)	996.35 ± 310.1	-
Adiponectin (ng/mL)	7219.18 ± 3479.9	-

*Only the first 5 variables listed in the table were measured 3.5 hours after the postprandial lipemia challenge in GOLDN
